# *Peste des Petits Ruminants* Virus Enters Caprine Endometrial Epithelial Cells via the Caveolae-Mediated Endocytosis Pathway

**DOI:** 10.3389/fmicb.2018.00210

**Published:** 2018-02-15

**Authors:** Bo Yang, Xuefeng Qi, Hui Guo, Peilong Jia, Shuying Chen, Zhijie Chen, Ting Wang, Jingyu Wang, Qinghong Xue

**Affiliations:** ^1^China Institute of Veterinary Drug Control, Beijing, China; ^2^College of Veterinary Medicine, Northwest A&F University, Xianyang, China

**Keywords:** PPRV, entry, caprine endometrial epithelial cells, caveolin, endocytosis

## Abstract

*Peste des petits ruminants* virus (PPRV) causes an acute and highly contagious disease of sheep and goats and has spread with alarming speed around the world. The pathology of *Peste des petits ruminants* is linked to retrogressive changes and necrotic lesions in lymphoid tissues and epithelial cells. However, the process of PPRV entry into host epithelial cells remains largely unknown. Here, we performed a comprehensive study of the entry mechanism of PPRV into caprine endometrial epithelial cells (EECs). We clearly demonstrated that PPRV internalization was inhibited by chloroquine and ammonium chloride, which elevate the pH of various organelles. However, PPRV entry was not affected by chlorpromazine and knockdown of the clathrin heavy chain in EECs. In addition, we found that the internalization of PPRV was dependent on dynamin and membrane cholesterol and was suppressed by silencing of caveolin-1. Macropinocytosis did not play a role, but phosphatidylinositol 3-kinase (PI3K) was required for PPRV internalization. Cell type and receptor-dependent differences indicated that PPRV entry into caprine fetal fibroblast cells (FFCs) occurred via a different route. Taken together, our findings demonstrate that PPRV enters EECs through a cholesterol-dependent caveolae-mediated uptake mechanism that is pH-dependent and requires dynamin and PI3K but is independent of clathrin. This potentially provides insight into the entry mechanisms of other morbilliviruses.

## Introduction

*Peste des petits ruminants* (PPR) is a severe infectious disease of goats and sheep. In 1979, PPR virus (PPRV) was classified as a *Morbillivirus* under the family *Paramyxoviridae* and the order *Mononegavirales* (Gibbs et al., 1979). The life cycle of PPRV is 6–8 h in permissive cells (Kumar et al., 2013). Like all morbilliviruses, PPRV has an established lymphatic and epithelial tropism (Couacy-Hymann et al., 2007; Hammouchi et al., 2012). Signaling lymphocyte activation molecule (SLAM) is a member of the C2 subset of the immunoglobulin superfamily exclusively expressed on immune cells but not epithelial cells and has been identified as a receptor for morbilliviruses (Tatsuo et al., 2000; Tatsuo et al., 2001; Baron, 2005). Nectin-4 is mainly expressed in epithelial tissues and encoded by multiple haplotypes in different sheep breeds around the world (Birch et al., 2013). Recently, it was identified as an epithelial receptor for measles virus (MeV), canine distemper virus, phocine distemper virus and PPRV, and this has shed light on the mode of entry of these viruses (Muhlebach et al., 2011; Noyce et al., 2011; Pratakpiriya et al., 2012; Melia et al., 2014).

Enveloped viruses enter the cell through two pathways: direct fusion and receptor-mediated endocytosis. The majority of Paramyxoviruses enter host cells via fusion between the viral envelope and the cell membrane. Fusion is attributed to the interaction between the HR1 and HR2 domains of the F protein, leading to close proximity between the viral and host cell membranes (Lee et al., 2007; Muhlebach et al., 2008). However, it has been shown previously that MeV enters Vero cells that express SLAM and PVRL4 using a receptor-mediated macropinocytosis-like pathway (Delpeut et al., 2017). Moreover, a recent study demonstrated that SLAM can also mediate MeV endocytosis (Goncalves-Carneiro et al., 2017). However, MeV enters target cells via membrane fusion at the cell surface in most cases, a process limited to viruses that can be endocytosed and activate type I interferon (Hornung et al., 2004).

Most animal viruses enter host cells via endocytic pathways, which include macropinocytosis, phagocytosis, and clathrin- and caveolae-dependent and -independent pathways (Sieczkarski and Whittaker, 2002; Conner and Schmid, 2003; Pelkmans and Helenius, 2003; Marsh and Helenius, 2006). Different families of viruses may utilize different endocytic pathways (Mercer and Helenius, 2009; Mercer et al., 2010; Nicola et al., 2013), the major one being clathrin-mediated endocytosis used by viruses such as hepatitis C virus (Min et al., 2017), African swine fever virus (Galindo et al., 2015), Dengue virus (Acosta et al., 2009), Singapore grouper iridovirus (Wang et al., 2014), human papillomavirus type 16 (Schelhaas et al., 2012), simian hemorrhagic fever virus (Cai et al., 2015), egg drop syndrome virus (Huang et al., 2015) and Hantaan virus (Jin et al., 2002). Previous studies indicated that HIV uses dynamin-dependent endocytosis during cell-to-cell transmission (Miyauchi et al., 2009; Sloan et al., 2013). Caveolae-mediated endocytosis is the second most prevalent pathway used by Ebola virus, simian virus 40 and Japanese encephalitis virus to enter cells (Anderson et al., 1996; Empig and Goldsmith, 2002; Zhu et al., 2012). Accumulating evidence indicates that many viruses can infect different target cells via existing uptake pathways rather than through unique mechanisms (Cantin et al., 2007; Cosset and Lavillette, 2011; Rahn et al., 2011; Han et al., 2016). In addition, vaccinia virus (Mercer and Helenius, 2008), Ebola virus (Nanbo et al., 2010; Saeed et al., 2010), influenza virus (de Vries et al., 2011; Rossman et al., 2012), adenovirus type 35 (Kalin et al., 2010), and picornaviruses such as echovirus 1 (Krieger et al., 2013) and coxsackievirus B (Coyne et al., 2007), enter cells via macropinocytosis. Recent studies demonstrated that paramyxoviruses including Nipah virus, Sendai virus, human metapneumovirus, human respiratory syncytial virus, Newcastle disease virus and MeV (Cantin et al., 2007; Kolokoltsov et al., 2007; Diederich et al., 2008; Pernet et al., 2009; Schowalter et al., 2009; Goncalves-Carneiro et al., 2017), utilize the endocytic machinery for entry. Furthermore, virus entry may involve various factors that are cell or virus type dependent, such as dynamin, cholesterol, Na^+^/H^+^ exchangers, phosphatidylinositol 3 kinase (PI3K) and acidic pH (Nicola et al., 2003; Cantin et al., 2007; Mercer and Helenius, 2009; Kalin et al., 2010; Zhu et al., 2012; Sloan et al., 2013).

The above findings suggest that entry of the paramyxovirus PPRV into cells may use endocytosis as an additional mechanism. In the present study, we investigate the mechanism of entry of PPRV into caprine endometrial epithelial cells (EECs). We used a variety of chemical inhibitors and an RNA interference (RNAi) approach to target cellular molecules involved in the PPRV entry process. Our data demonstrated that the entry of PPRV into caprine EECs occurred via the caveolae-mediated endocytosis pathway, which differed from the entry mechanism into caprine fetal fibroblasts cells. Collectively, our data support the idea that in addition to direct fusion at the cell surface, PPRV may penetrate cells through endocytosis, at least in goat epithelial cells.

## Materials and Methods

### Cell Lines and Viruses

Endometrial epithelial cell were immortalized by transfection with human telomerase reverse transcriptase, and we confirmed that their secretory function was consistent with that of primary EECs (Qi X. et al., 2012; Qi X.F. et al., 2012; Wang et al., 2015) kindly provided by Prof. Yaping Jin (Northwest A&F University Yangling, Shaanxi, China). As a result of immortalization, a portion of the EECs were prone to aging and became larger and longer than primary cells with increased passage. Caprine fetal fibroblast cells (FFCs), from our laboratory culture collection, were cultured in Dulbecco’s Minimal Essential Medium/Nutrient Mixture F-12 Ham’s medium (DMEM/F12) supplemented with 10% fetal bovine serum (FBS; Gibco, Carlsbad, CA, United States), 100 IU/ml of penicillin and 10 μg/ml of streptomycin at 37°C in 5% CO_2_. The PPRV vaccine strain, Nigeria 75/1, was also from our laboratory culture collection. All experimental materials were autoclaved at 110°C for 20 min to kill PPRV when each experiment was completed. Our laboratory conformed to P2 (BSL-2) laboratory requirements. All experiments were completed under P2 laboratory conditions.

### Inhibitors, Antibodies, siRNAs and Plasmids

Chlorpromazine (CPZ), methyl-β-cyclodextrin (MβCD), ammonium chloride (NH_4_Cl), chloroquine, dynasore, wortmannin, nystatin and 5-ethyl-*N*-isopropyl amiloride (EIPA) were purchased from Sigma (St. Louis, MO, United States). Cholera toxin B (CTxB) and transferrin (TF) conjugated to Alexa Fluor 594 were purchased from Invitrogen (Carlsbad, CA, United States).

Anti-PPRV-N monoclonal antibody was provided by the China Animal Health and Epidemiology Center (Qingdao, China). Rabbit polyclonal anti-PPRV-H antibody was generated by our laboratory. Specific antibodies against caveolin-1 were purchased from Santa Cruz Biotechnology (Santa Cruz, CA, United States). Anti-clathrin monoclonal antibody and anti-nectin-4 monoclonal antibody was purchased from Abcam (Cambridge, MA, United States). Anti-β-actin antibody, HRP-conjugated secondary antibodies, and FITC-conjugated anti-mouse and anti-rabbit IgG were purchased from Transgen Biotechnology (Beijing, China). TRITC-phalloidin was purchased from Sigma.

Pooled and validated siRNAs targeting caveolin-1 (Cav-1) were purchased from Santa Cruz Biotechnology. ShRNAs targeting clathrin heavy-chain (CHC) (shCHC) and scramble RNAs were kindly provided by Dr. Hung-Jen Liu (Institute of Molecular Biology, National Chung Hsing University, Taichung, Taiwan).

### Cell Viability Assay

Briefly, EECs and FFCs were seeded in 96-well cell culture plates with 200 μl DMEM/F12 containing 2% FBS at a density of 1 × 10^4^ cells per well. After incubation at 37°C in 5% CO_2_ for 24 h, different pharmacological inhibitors at the indicated concentrations were added and incubated for 24 h. Twenty microliters of MTT solution (Sigma–Aldrich Co.) was subsequently added and incubated for 4 h to allow the MTT to be fully metabolized. Then, the medium was removed and the cells were washed with DMEM/F12. Finally, cells were resuspended in formazan in 100 μl of DMSO solution and the optical density was read at 540 nm.

### Pharmacological Inhibition Treatment and Viral Infection Assays

To determine the effects of chemical inhibitors on viral entry and the post-entry steps, cells were treated with different inhibitors 1 h before or after PPRV inoculation. Briefly, monolayers of EECs or FFCs grown in 24-well plates were pretreated with the respective inhibitors at the indicated concentrations at 37°C for 1 h. Cell viability upon drug treatment was determined by a cell viability assay. Cells were then inoculated with PPRV at a multiplicity of infection (MOI) of 10 at 4°C for 1 h. After adsorption, the medium was discarded and the cells were subsequently washed with phosphate-buffered saline (PBS). Then, cells were cultured in maintenance medium containing 2% serum at 37°C, in the continuous presence of the inhibitors. DMSO-treated cells were used as a control. At the indicated time points, cells were collected for western blot analysis and further prepared for confocal laser scanning microscopy (CLSM) analysis, whereas supernatants were harvested for virus titration.

### Transfection and Gene Silencing

Endometrial epithelial cells grown to 80% confluence in 6-well cell culture plates were transfected with 4 μg/well of CHC shRNA plasmids or 300 nM Cav-1 siRNA using Turbofect transfection reagent (Thermo Fisher Scientific). Then, the cells were incubated in opti-MEM (Gibco) medium at 37°C for 48 h. The reaction mixture was discarded and cells were then infected with PPRV at a MOI of 10. At 12 h post-infection (hpi), the cells were collected for western blot analysis and viral titration. The silencing efficiencies were quantified by western blot analysis.

### Transmission Electron Microscopy (TEM)

Ultra-thin sections (70 nm) of cells were prepared and examined under a Hitachi HT-7700 transmission electron microscope (Hitachi High Technologies Co., Japan) as described previously (Ishii et al., 2010; Huang et al., 2015). Briefly, PPRV was inoculated into EECs cultured in a Cell Factory system (Nunc). At 5 days post-infection, cells and culture medium were harvested, subjected to three cycles of freeze-thawing, and then centrifuged at 1500 × *g* for 10 min. Subsequently, the supernatant was centrifuged at 20,000 rpm for 120 min at 4°C in a SW41Ti rotor (Beckman Inc.). Pelleted virions were resuspended in 1 ml of DMEM/F12. Briefly, monolayers of EECs were infected with PPRV at a MOI of 50. After adsorption at 4°C for 1 h, the samples were shifted to 37°C for 15 min, 30 min, 1 h and 2 h. Cells were then collected by centrifugation at 800 × *g* for 5 min for subsequent sample preparation.

### Confocal Laser Scanning Microscopy

Endometrial epithelial cells were grown on coverslips to a confluency of ∼60%, and infected with PRRV at a MOI of 20. At the indicated times post-infection, cells were washed four times with PBS and fixed in 4% paraformaldehyde. Cells were then washed four times again with PBS and treated with 0.1% Triton X-100 for 15 min. Cells were then incubated with 1% bovine serum albumin (BSA) and appropriate primary antibodies for 1 h at 37°C. Then, cells were washed and further incubated with FITC- or TRITC-conjugated secondary antibodies. Actin filaments were stained with TRITC-phalloidin (2 μg/ml) for 40 min at 25°C. Finally, cells were treated with 1 μg/ml of DAPI solution for 15 min and analyzed by confocal microscopy (CLSM Leica SP8, Germany). In addition, 3D images were recorded using a Leica TCS SP8 laser-scanning confocal microscope and analyzed with Imaris software (Bitplane) for visualization, manual segmentation and surface rendering.

To further investigate endocytosis, EECs were incubated with 10 μg/ml Alexa Fluor 594-TF for 15 min at 37°C. Cells were washed with acidic buffer (0.2 M acetic acid, 0.5 M NaCl, pH 2.5) for 5 min to remove surface-bound TF. For the CTxB uptake assay, cells were incubated with 20 μg/ml Alexa Fluor 594-CTxB for 45 min at 37°C. After washing with PBS, cells were fixed in 4% paraformaldehyde. Finally, TF and CTxB internalization was examined by CLSM.

### Western Blot Analysis

At the indicated time points, cell lysates were generated by adding 5 × sodium dodecyl sulfate-polyacrylamide gel electrophoresis (SDS-PAGE) sample buffer to the cells and boiling for 10 min. After centrifugation, protein samples were separated on 12% SDS-PAGE gels and then transferred onto 0.22-μm polyvinylidene difluoride membranes (Millipore, Billerica, MA, United States). Membranes were then blocked with 5% non-fat milk solution containing 0.05% Tween-20 at 4°C overnight and subsequently incubated for 2 h with primary antibodies. After washing, the membranes were reacted with HRP-conjugated secondary antibodies. Bound antibodies were detected with ECL immunoblotting detection reagents (Millipore). Densitometry analysis using Image J2^∗^ software was conducted for protein quantification.

### Virus Titration

Collected culture supernatants were centrifuged to remove cell debris. Briefly, EECs cultivated in 96-well plates were inoculated with virus dilutions (100 μL/well) prepared by 10-fold serial dilutions. Cells were incubated at 37°C with 5% CO_2_ for about 120 h, and any cytopathic effect (CPE) was recorded. Virus titers were calculated using the Reed–Muench method, and recorded as the TCID_50_/mL. Each test was performed in triplicate.

### Statistical Analyses

Statistical analysis was performed using the GraphPad Prism version 6.0 (GraphPad Software, San Diego, CA, United States) software. The two-tailed, unpaired Student *t*-test was used to determine statistical significance between the groups. A value of *P* < 0.05 was considered to indicate statistical significance, whereas *P* < 0.01 was considered to indicate a highly significant difference. All data were expressed as the mean ± standard (SD), and normalized against the mean of the control group from at least three independent experiments.

## Results

### Kinetics of PPRV Entry and Replication in Cells

**FIGURE 1:**
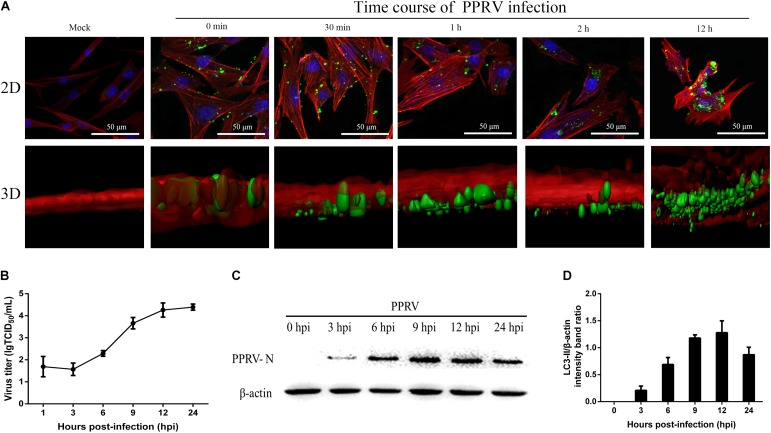
Kinetics of PPRV entry and replication in EECs. **(A)** CLSM analysis of actin filaments (red), viral particles (green) and cell nuclei (blue) in PPRV-infected EECs at the indicated time points post-infection. 3D images are shown in the lower panels. **(B)** One-step growth curve of PPRV in EECs, as determined by a TCID_50_ assay. **(C,D)** Western blot analysis of N protein expression to indicate virus replication in PPRV-infected EECs at the indicated time points post-infection. Representative blots and densitometry analysis of the data are presented in **(C,D)**, respectively. β-actin was used as an internal control. The bars indicate the mean ± SD from three independent experiments. SD, standard deviation.

To investigate PPRV entry and its replication cycle in EECs, initial experiments were performed to analyze the uptake of PPRV into EECs at the indicated time points using CSLM. As shown in **Figure 1A**, viral particles were internalized and distributed within the boundaries of the cells. Viruses distributed around the nuclei over time, as shown by the gradual intensification of the green fluorescent signal specific to PPRV-N, which peaked at 12 hpi. Moreover, 3D images showed that PPRV was adsorbed onto the cell surface but not inside the cells at 0 min. PPRV appeared in the lower layer of the cell membrane over time. PPRV moved inside the EECs at 1 hpi. Importantly, the 3D surface rendering data demonstrated that PPRV was located on the cell membrane as indicated by an intense green signal in the 2D image. Furthermore, because the cell membrane is in the upper layer, PPRV was successfully internalized into the EECs as indicated by a weak, dark green signal in the 2D image. The viral titer increased rapidly from 3 hpi, peaking at 12 hpi (**Figure 1B**). Our results also showed that N proteins were detectable from 3 hpi, then sharply increased, reaching a peak at 12 hpi (**Figures 1C,D**). Analysis by CLSM, along with N protein expression and viral titration data, suggested that the life cycle of PPRV is 9–12 h in cultured host epithelial cells. Therefore, 12 hpi was considered as the optimal time point in subsequent experiments for determining the viral protein expression levels and titers.

### PPRV Entry Depends on Low pH and Dynamin

**FIGURE 2:**
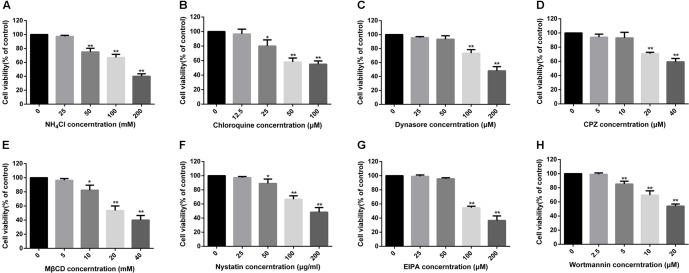
MTT assays to determine the cytotoxicity of inhibitors in EECs. **(A–H)** All tested inhibitors were subjected to an MTT assay to evaluate their cytotoxicity toward EECs. Cells were exposed to the indicated concentrations of inhibitors for 24 h. The bars indicate the mean ± SD from three independent experiments. SD, standard deviation; ^∗^*P* < 0.05; ^∗∗^*P* < 0.01.

**FIGURE 3:**
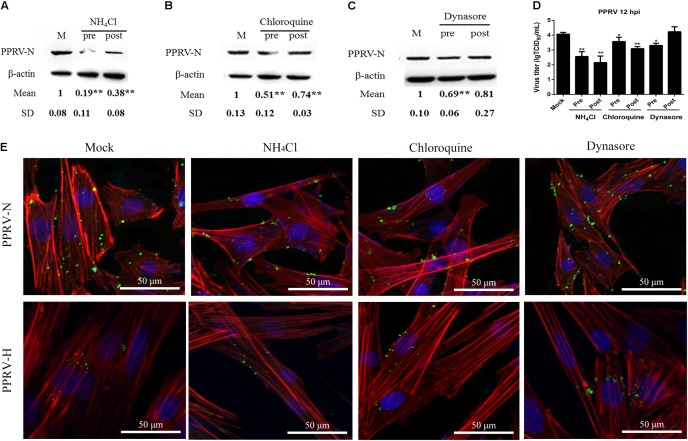
*Peste des petits ruminants* virus (PPRV) entry depends on low pH and dynamin. **(A–C)** Western blot analysis of the entry of PPRV into mock-, NH_4_Cl-, chloroquine- or dynasore-treated cells. β-actin was used as an internal control. **(D)** Virus titration analysis of the entry and replication of PPRV in NH_4_Cl-, chloroquine- or dynasore-treated cells. **(E)** CLSM analysis of TRITC-phalloidin (red), anti-PPRV (green) and DAPI (blue) in PPRV-infected EECs pre-treated with NH_4_Cl, chloroquine or dynasore. The bars indicate the mean ± SD from three independent experiments. SD, standard deviation; ^∗^*P* < 0.05; ^∗∗^*P* < 0.01.

To confirm whether PPRV entry into EECs is pH dependent, we detected the effect of two chemical inhibitors: chloroquine, a cellular endosome acidification inhibitor (Silva et al., 2007), and NH_4_Cl, a lysosomotropic weak base that immediately raises the pH of acidic vesicles (Mizzen et al., 1985). NH_4_Cl treatment at 25 mM and chloroquine treatment at 12.5 μM did not affect cell viability (**Figures 2A,B**). The expression level of PPRV-N protein was analyzed by densitometry, and normalized against β-actin. NH_4_Cl treatment before or after PPRV addition significantly blocked the entry and replication of PPRV (1.00 ± 0.08, 0.19 ± 0.11, 0.38 ± 0.08 for mock, pre- and post-treatment samples, respectively) (**Figure 3A**). Chloroquine treatment before or after PPRV addition also significantly inhibited the entry and replication of PPRV (1.00 ± 0.13, 0.51 ± 0.12, 0.74 ± 0.03 for mock, pre- and post-treatment samples, respectively) (**Figure 3B**). Virus titration analysis indicated that NH_4_Cl-treated and chloroquine-treated cells showed inhibited PPRV entry and replication (**Figure 3D**). Similarly, a more intense green fluorescent signal specific to PPRV-N and PPRV-H was observed on the membrane of cells treated with NH_4_Cl and chloroquine compared with control cells (**Figure 3E**). Therefore, PPRV entry was strongly inhibited in NH_4_Cl-treated and chloroquine-treated cells. These results demonstrated that PPRV infection requires low pH conditions, but further investigation will be necessary to uncover the mechanism of action of NH_4_Cl on virus entry and replication.

Dynamin is required for the mediation of newly formed vesicles in the caveolin- and clathrin-dependent endocytic pathways (Praefcke and McMahon, 2004). To determine the effects of dynamin on PPRV infection, dynasore was used to treat EECs for the indicated time periods. Dynasore treatment at 50 μM did not affect cell viability (**Figure 2C**). However, dynasore treatment before PPRV inoculation significantly inhibited PPRV infection in EECs (1.00 ± 0.10, 0.69 ± 0.06, 0.81 ± 0.27 for mock, pre- and post-treatment samples, respectively) (**Figure 3C**). Virus titration analysis indicated that dynasore treatment did not influence PPRV replication (**Figure 3D**). However, dynasore treatment did induce the expression of green fluorescent signals specific to the PPRV-N and PPRV-H proteins on the cell membrane that were not observed with control cells (**Figure 3E**). Therefore, PPRV entry was inhibited in dynasore-treated cells. These results suggested that dynamin is critical for PPRV entry into cells.

### Clathrin Is Not Required for PPRV Entry into EECs

**FIGURE 4:**
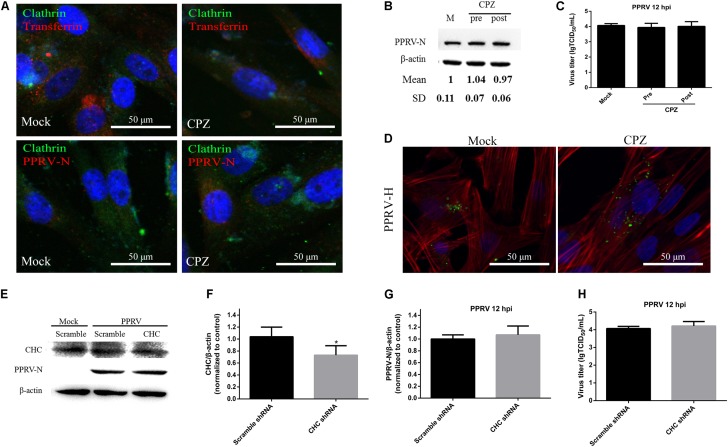
CME is not the pathway for PPRV entry into EECs. **(A–C)** Effect of CPZ treatment on PPRV entry and infection. **(A)** CLSM analysis of clathrin (green), viral particles (red) and cell nuclei (blue) in PPRV-infected EECs pre-treated with CPZ. The effect of CPZ on Alexa Fluor 594–TF uptake was used as a positive control (red fluorescence). **(B)** Western blot analysis of the entry of PPRV into CPZ-treated cells. β-actin was used as an internal control. **(C)** Virus titration results of PPRV in CPZ -treated cells. **(D)** CLSM analysis of TRITC-phalloidin (red), anti-PPRV (green) and DAPI (blue) in PPRV-infected EECs pre-treated with CPZ. **(E)** Western blot analysis of the entry of PPRV into shRNA-transfected cells. **(F)** Efficiency of CHC downregulation was analyzed by immunoblotting. **(G)** The relative quantification of viral proteins was determined by densitometry against β-actin in shRNA-transfected cells. **(H)** Virus titration analysis of PPRV in shRNA-transfected cells. The bars indicate the mean ± SD from three independent experiments. SD, standard deviation; ^∗^*P* < 0.05; ^∗∗^*P* < 0.01.

To assess the role of clathrin in PPRV entry into EECs, CPZ was used to specifically block this pathway (Wang et al., 1993). CPZ was used at 10 μM based on the cell viability assay data (**Figure 2D**). For CLSM, EECs were pretreated with CPZ and this was maintained during PPRV infection. Transferrin (TF) was used as a positive control in the clathrin-mediated endocytosis pathway. As shown in **Figure 4A**, red fluorescence was significantly reduced in the presence of CPZ, confirming that CPZ effectively blocks the uptake of TF via the clathrin-medicated endocytosis pathway, whereas no effect was observed with PRRV-infected cells. Western blot analysis and a viral titration assay were performed on EECs treated with CPZ at 1 h either before or after PPRV inoculation, and CPZ was maintained during infection. The expression level of PPRV-N protein was analyzed by densitometry, and normalized against β-actin. Less pronounced changes were detected in the normalized viral N protein expression levels (1.00 ± 0.11, 1.04 ± 0.07, 0.97 ± 0.06 for mock, pre- and post-treatment samples, respectively) and viral titers (LgTCID_50_/ml) (4.07 ± 0.12, 3.94 ± 0.27, 4.01 ± 0.31, for the different samples, respectively) (**Figures 4B,C**). Moreover, CPZ treatment did not induce the expression of a green fluorescent signal specific to PPRV-H on the cell membrane when compared with control cells (**Figure 4D**). These findings indicated that CPZ treatment did not affect PPRV entry and replication in cells.

To further confirm the role of clathrin in PPRV entry and replication, we detected the expression levels of CHC and PPRV-N by inhibiting CHC expression with shRNA (**Figure 4E**). Based on western blot analysis, the expression levels of CHC were found to be significantly downregulated by the specific shCHC compared with the Scramble control (1.14 ± 0.17, 0.67 ± 0.16, for Scramble and shCHC samples, respectively, *P* < 0.05) (**Figure 4F**). When transfected with shCHC prior to PPRV addition, the relative expression level of PPRV-N protein was not changed significantly, as determined by western blot analysis (1.00 ± 0.07, 1.07 ± 0.15, for Scramble and shCHC samples, respectively) (**Figure 4G**). The virus titration data (4.07 ± 0.12, 4.21 ± 0.25, for Scramble and CHC siRNA samples, respectively) also supported the finding that downregulation of CHC expression did not significantly influence PPRV replication and release (**Figure 4H**).

### PPRV Entry Depends on Caveolin and Requires Plasma Membrane Cholesterol

**FIGURE 5:**
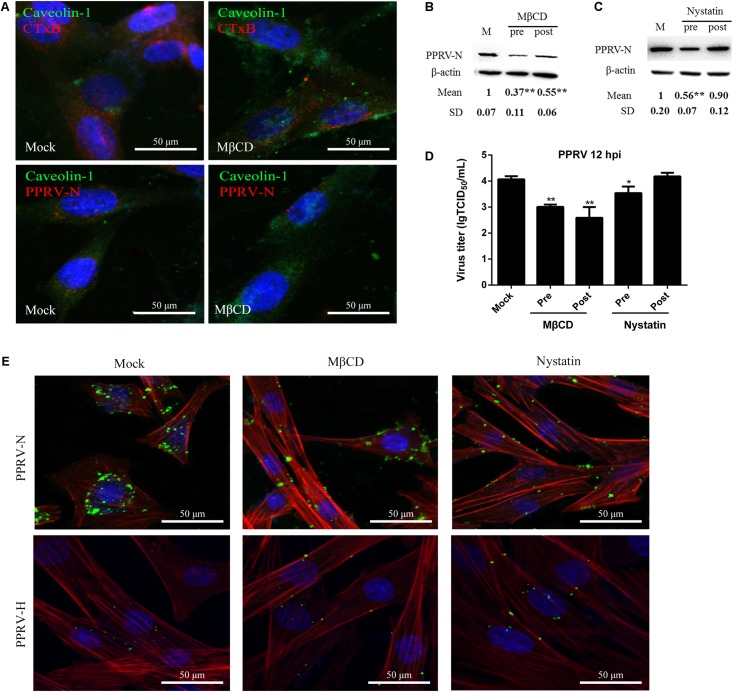
*Peste des petits ruminants* virus entry and replication in EECs requires plasma membrane cholesterol. **(A)** CLSM analysis of caveolin-1 (green), viral particles (red) and cell nuclei (blue) in PPRV-infected EECs pre-treated with MβCD. The effect of MβCD on Alexa Fluor 594–CTxB uptake was apparent (red). **(B,C)** Western blot analysis of the entry of PPRV in mock-, MβCD- or nystatin-treated cells. β-actin was used as an internal control. **(D)** Virus titration analysis of PPRV treated with MβCD and nystatin. **(E)** CLSM analysis of TRITC-phalloidin (red), anti-PPRV (green) and DAPI (blue) in PPRV-infected EECs pre-treated with MβCD and nystatin. The bars indicate the mean ± SD from three independent experiments. SD, standard deviation; ^∗^*P* < 0.05; ^∗∗^*P* < 0.01.

Membrane cholesterol is required for the formation of caveolae and is an essential component of lipid rafts. Depletion of cholesterol from the membrane with MβCD or sequestration of cholesterol with nystatin can significantly block caveolae-mediated endocytosis (Pitha et al., 1988; Sanchez-San Martin et al., 2004). In the present study, MβCD treatment at 5 mM and nystatin treatment at 25 μg/ml did not affect cell viability (**Figures 2E,F**). Therefore, these concentrations were chosen for the next set of experiments. Cholera toxin B (CTxB) was used as a positive control in the caveolin-dependent pathway. As shown in **Figure 5A**, MβCD treatment could effectively block uptake of CTxB via the caveolin-mediated endocytosis pathway. Importantly, MβCD treatment also significantly inhibited PPRV internalization (**Figure 5A**). Western blot analysis showed that MβCD treatment before and after virus addition significantly inhibited the entry and replication of PPRV (1.00 ± 0.07, 0.37 ± 0.11, 0.55 ± 0.06 for mock, pre- and post-treatment samples, respectively) (**Figure 5B**). Furthermore, nystatin treatment before virus addition significantly inhibited the virus entry. However, nystatin treatment after virus addition did not affect PPRV replication in EECs (**Figure 5C**). Virus titration analysis indicated that MβCD treatment significantly influenced PPRV entry and replication. However, nystatin treatment showed no impact on PPRV replication (**Figure 5D**). CLSM revealed that nystatin and, in particular, MβCD treatment induced green fluorescence signals specific to PPRV-N and PPRV-H on the cell membrane that were not evident in control cells (**Figure 5E**). Therefore, PPRV entry was obviously blocked in MβCD and nystatin-treated cells. These data indicate that PPRV entry into cells depends on caveolae and requires the involvement of cholesterol.

**FIGURE 6:**
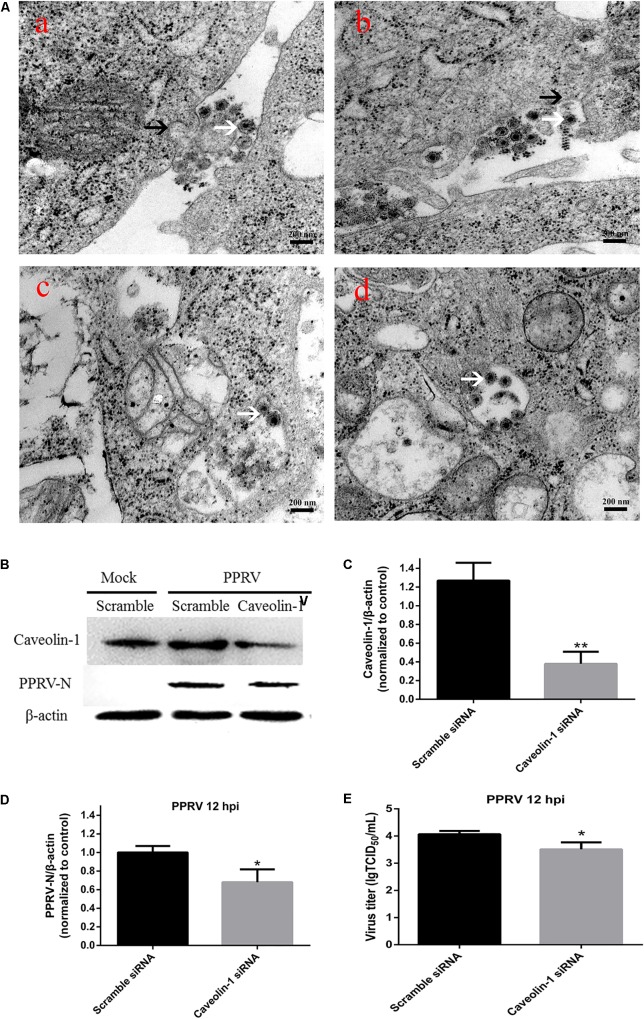
*Peste des petits ruminants* virus entry into EECs depends on caveolae-mediated endocytosis. **(A)** Microscopic analysis of PPRV uptake into EECs. Monolayers of EECs grown on tissue culture plates were infected with PPRV at a MOI of 50. After adsorption at 4°C for 1 h, the samples were shifted to 37°C for 15 min **(a)**, 30 min **(b)**, 1 h **(c)** and 2 h **(d)**. Transmission electron microscopy image showing the localization of PPRV-induced plasma membrane invagination, as indicated by black arrows. Virions are indicated by white arrows. **(B)** Western blot analysis of the entry of PPRV into siRNA-transfected cells. **(C)** Efficiency of caveolin downregulation was analyzed by immunoblotting. **(D)** The relative quantification of viral proteins as determined by densitometry against β-actin in siRNA-transfected cells. **(E)** Virus titration analysis of PPRV in siRNA-transfected cells. The bars indicate the mean ± SD from three independent experiments. SD, standard deviation; ^∗^*P* < 0.05; ^∗∗^*P* < 0.01.

PPRV virions are pleomorphic in shape and vary in size from 150 to 700 nm (Gibbs et al., 1979; Kumar et al., 2014). In the present work, enveloped PPRV particles of about 150–200 nm were initially observed attached to the EEC surface at 15 min post infection (**Figure 6A-a**). No larger particles were observed, probably due to the methods of virus purification and sample production. PPRV triggered plasma membrane invagination forming a cave-like structure wrapped around the bound viruses at 30 min post infection (**Figure 6A-b**). The vesicle then traveled to the cytoplasm after 1 hpi (**Figures 6A-c,d**). To further confirm the role of Cav-1 in PPRV entry and replication, we detected the expression levels of Cav-1 and PPRV-N by inhibiting Cav-1 expression with siRNA (**Figure 6B**). Based on western blot analysis, the expression levels of Cav-1 were significantly downregulated by specific siCav-1 (1.27 ± 0.19, 0.38 ± 0.13, for Scramble and siCav-1 samples, respectively, *P* < 0.01) (**Figure 6C**). During siCav-1 treatment prior to PPRV addition, the expression of PPRV-N protein was significantly decreased from 1.00 ± 0.07 to 0.68 ± 0.14 for the Scramble and Cav-1 siRNA samples, respectively (**Figure 6D**). Virus titration analysis indicated that downregulation of Cav-1 expression significantly impaired PPRV replication and release (4.07 ± 0.12, 3.51 ± 0.26, for the Scramble and Cav-1 siRNA samples, respectively) (**Figure 6E**).

### PPRV Entry Does Not Depend on Macropinocytosis but Requires PI3K

**FIGURE 7:**
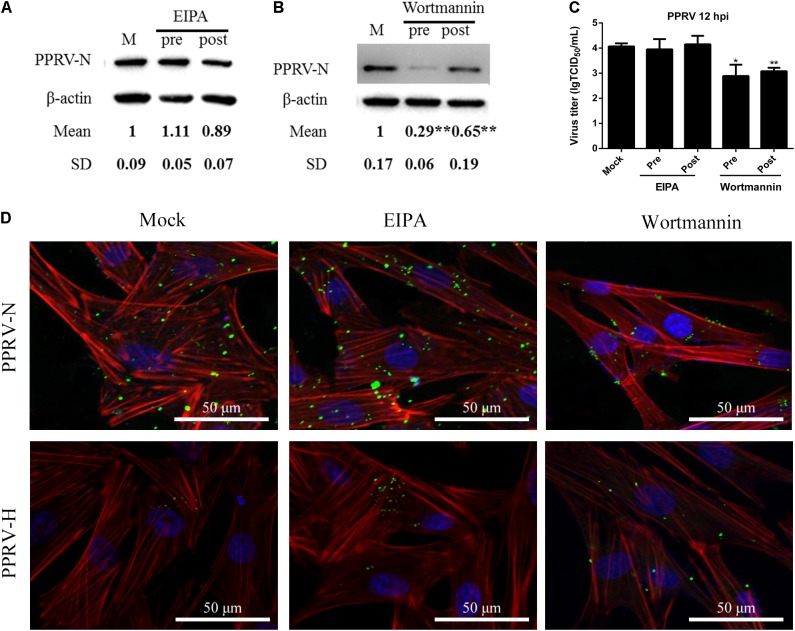
*Peste des petits ruminants* virus entry does not depend on macropinocytosis but requires PI3K. **(A,B)** Western blot analysis of viral protein expression in mock-, EIPA- or wortmannin-treated cells. Cells were treated with EIPA and wortmannin 1 h before (Pre) or 1 h after virus addition (Post) and this was maintained during infection. After 12 hpi (PPRV, MOI 10), equivalent amounts of protein were analyzed by western blot using an anti-PPRV-N antibody. β-actin was used as an internal control. **(C)** Virus titration analysis of PPRV treated with EIPA and wortmannin. **(D)** CLSM analysis of TRITC-phalloidin (red), anti-PPRV (green) and DAPI (blue) in PPRV-infected EECs pre-treated with EIPA and wortmannin. The bars indicate the mean ± SD from three independent experiments. SD, standard deviation; ^∗^*P* < 0.05; ^∗∗^*P* < 0.01.

Macropinosome formation is dependent on Na^+^/H^+^ exchangers (NHE), and EIPA is a specific inhibitor of NHE (Koivusalo et al., 2010; Mercer and Helenius, 2012). To investigate whether PPRV entry involves macropinocytosis, PPRV was added to EECs pretreated with DMSO or EIPA. EIPA treatment at 50 μM did not affect cell viability (**Figure 2G**). Both western blot and viral titration analysis demonstrated that EIPA treatment before and after virus addition did not inhibit the entry or replication of PPRV significantly (**Figures 7A,C**). EIPA treatment did not induce the expression of green fluorescent signals specific to the PPRV-N and PPRV-H proteins on the cell membrane when compared with control cells (**Figure 7D**). These findings indicated that EIPA treatment did not affect PPRV entry and replication. Therefore, PPRV entry into EECs does not require NHE. In addition, it was reported that PI3K is involved in multiple stages of macropinocytosis (Mercer and Helenius, 2012). To further analyze whether PPRV internalization required PI3K, EECs were treated with wortmannin, a specific PI3K inhibitor (Lee et al., 2005). Wortmannin treatment at 2.5 μM did not affect cell viability (**Figure 2H**) but significantly inhibited the productive infection of PPRV as evidenced by PRRV N expression levels and viral titers (*P* < 0.01, except *P* < 0.05 for the viral titer of pre-treated cells) (**Figures 7B,C**). Wortmannin treatment obviously induced green fluorescent signals specific to the PPRV-N and PPRV-H proteins on the cell membrane when compared with control cells (**Figure 7D**), which indicated that wortmannin treatment significantly inhibited PPRV uptake. Collectively, our data suggested that PPRV entry was not required for NHE, but the inhibition of PI3K activity significantly reduced PPRV entry and replication.

### Effects of Inhibitors on PPRV Entry into FFCs

**FIGURE 8:**
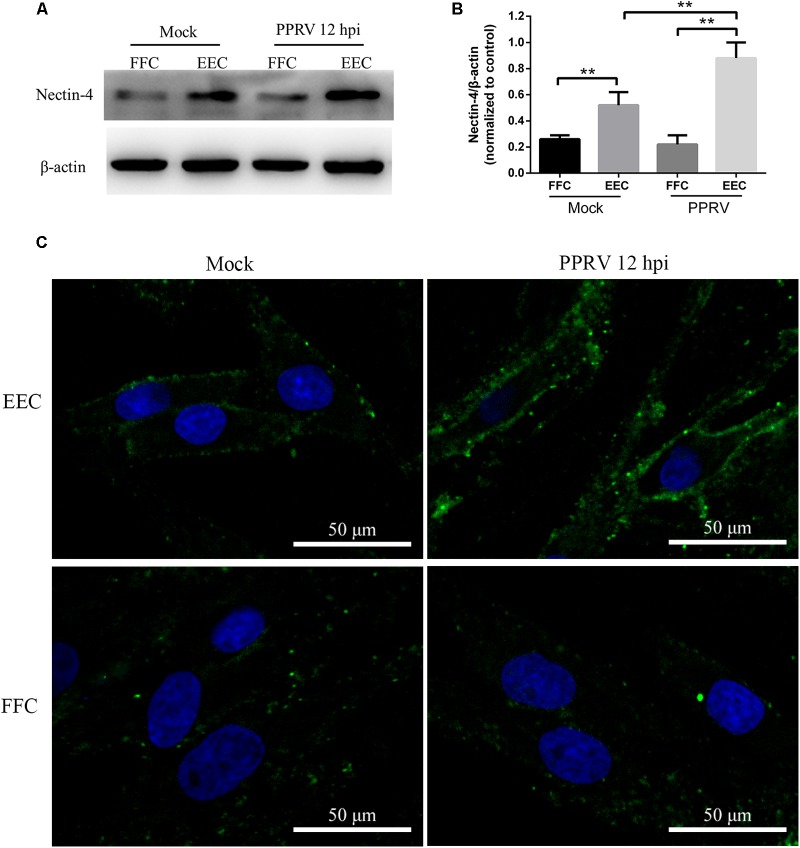
Receptor nectin-4 expression in EECs and FFCs inoculated with PPRV. **(A)** Western blot analysis of viral receptor expression in mock and PPRV infected cells. **(B)** The relative quantification of the receptor proteins was determined by densitometry against β-actin. **(C)** CLSM analysis of anti-nectin-4 (green) and DAPI (blue) in mock and PPRV infected cells. The bars indicate the mean ± SD from three independent experiments. SD, standard deviation; ^∗^*P* < 0.05; ^∗∗^*P* < 0.01.

**FIGURE 9:**
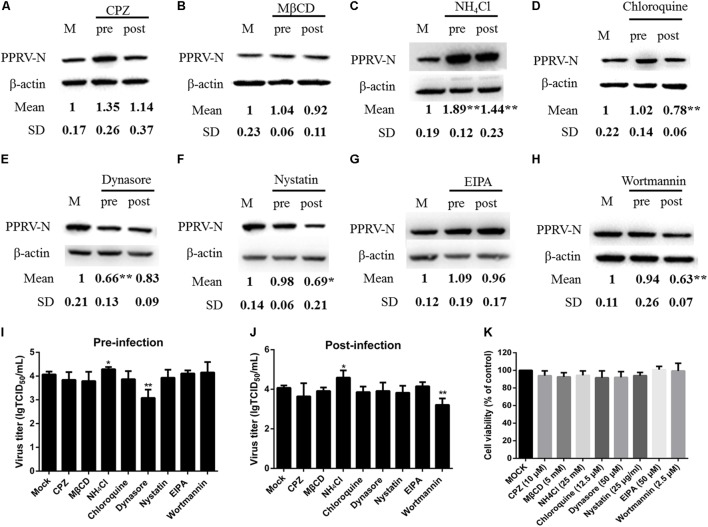
Effects of inhibitors on PPRV entry into FFCs. **(A–H)** Western blot analysis of the entry and replication of PPRV. Cells were treated with inhibitors 1 h before (Pre) or 1 h after virus addition (Post) and this was maintained during infection. After 12 hpi (PPRV, MOI 10), equivalent amounts of protein were analyzed by western blot with the anti-PPRV-N antibody, and β-actin was used as an internal control. The relative expression level was determined by densitometry. **(I,J)** Viral titration analysis of the entry and replication of PPRV in pre- and post-treatment samples. **(K)** The concentration of inhibitors did not affect cell viability in FFCs. All inhibitors were subjected to an MTT assay to evaluate their cytotoxicity toward FFCs that were exposed to the indicated concentrations for 24 h. The bars indicate the mean ± SD from three independent experiments. SD, standard deviation; ^∗^*P* < 0.05; ^∗∗^*P* < 0.01.

To compare PPRV entry into different types of host cells, we first investigated the expression level of the nectin-4 receptor in EECs and FFCs by western blot analysis (**Figure 8A**). Lower basal levels of nectin-4 were detected in FFCs compared with EECs (**Figure 8B**). Interestingly, our data showed that the amounts of nectin-4 protein were significantly increased in EECs but not in FFCs following PPRV infection (**Figure 8B**). Furthermore, CLSM revealed significantly enhanced staining of nectin-4 on the cell surface of EECs in response to PPRV infection (**Figure 8C**), whereas PPRV-infected FFCs exhibited diffuse staining of nectin-4 throughout the entire cytoplasm (**Figure 8C**). Importantly, pharmacological inhibition experiments using FFCs indicated that CPZ, MβCD and EIPA treatment before and after virus addition did not noticeably affect PPRV N protein expression or the viral titer in FFCs (**Figures 9A,B,G,I,J**), whereas NH_4_Cl treatment before or after PPRV addition resulted in a significant increase in N protein expression (**Figure 9C**), unlike the results obtained with EECs. Chloroquine, nystatin and wortmannin treatment prior to PPRV addition did not affect early entry of PPRV into FFCs; however, treatment post PPRV addition significantly blocked the replication of PPRV (**Figures 9D,F,H**). By contrast, dynasore treatment significantly inhibited the uptake of PPRV but not replication of the virus in FFCs (**Figure 9E**). Virus titration analysis further indicated that NH_4_Cl treatment significantly increased PPRV infection (**Figures 9I,J**), whereas dynasore treatment significantly blocked PPRV entry (**Figure 9I**) and wortmannin treatment significantly blocked PPRV replication (**Figure 9J**). Cell viability was not significantly affected by these chemical inhibitors in FFCs (**Figure 9K**). Overall, PPRV entry into FFCs may not depend on clathrin, caveolin, low pH, cholesterol, NHE and PI3K; however, dynamin plays an essential role. Nystatin and wortmannin treatment after PPRV addition significantly inhibited PPRV replication.

## Discussion

PPRV is an economically important pathogen that seriously constrains the productivity of small ruminants throughout the world. Clinically, PPR is characterized by a high fever, oculo-nasal discharge, diarrhea, dyspnea and sloughing of the epithelium of the oral and nasal mucosa (Balamurugan et al., 2014; Santhamani et al., 2016). It is worth noting that PPRV infection often causes fetal mummification, abortion late in pregnancy, or the birth of dead or weak lambs that die within the first few days (Borel et al., 2005; Abubakar et al., 2008). However, the pathogenesis of PPRV remains largely unknown, and is predominantly based on comparisons with morbilliviruses. Here we show that PPRV can successfully enter into EECs, further confirming that PPRV has epithelial tropism. In addition, the proliferation of PPRV in caprine EECs observed in the present study suggests that the clinical phenomenon of abortions in PPRV-infected goats may be attributed to the replication of PPRV in the uterus, thus providing a theoretical basis for the pathogenesis of PPRV.

Most animal viruses utilize the endocytic machinery for entry and replication in host cells. Viruses that enter target cells through endocytosis can easily pass through the plasma membrane barrier and the underlying cortical matrix (Han et al., 2016). Previous studies showed that paramyxoviruses enter into host cells via membrane fusion (Lee et al., 2007; Muhlebach et al., 2008). However, Newcastle disease virus may enter cells by caveolae-mediated endocytosis, a process dependent on an acidic pH (Cantin et al., 2007; Sanchez-Felipe et al., 2014). In addition, a recent study demonstrated that MeV particle internalization is independent of the clathrin-, caveolin- or dynamin-2-mediated endocytic pathways (Goncalves-Carneiro et al., 2017). Therefore, the pathway via which paramyxoviruses enter host cells is likely to be associated with cell type and the available receptors. For example, several studies have shown that SLAM plays an important role in facilitating the entry of morbilliviruses (Tatsuo et al., 2000, 2001; Baron, 2005; Goncalves-Carneiro et al., 2017). Several strains of morbillivirus were shown to use nectin-4 as a permissive receptor (Muhlebach et al., 2011; Noyce et al., 2011; Pratakpiriya et al., 2012; Sato et al., 2012; Melia et al., 2014). Interestingly, MeV enters breast and colon cancer cells through a PVRL4-mediated macropinocytosis pathway (Delpeut et al., 2017). Therefore, these studies concluded that morbilliviruses can enter target cells via different receptors, thereby triggering different signaling cascades (Hashimoto et al., 2002; Fujita et al., 2007; Noyce et al., 2011; Pratakpiriya et al., 2012; Birch et al., 2013; Delpeut et al., 2017; Goncalves-Carneiro et al., 2017). In this study, we first showed that PPRV could utilize caveolin-mediated endocytic pathways to enter epithelial cells. We also demonstrated that acidic pH, dynamin, cholesterol and PI3K are critical for the entry process. Although few morbilliviruses have been reported to enter cells in this manner, an increasing number of enveloped viruses have been reported to take advantage of caveolae-mediated endocytosis in a pH- and cholesterol-dependent manner, such as Newcastle disease virus (Cantin et al., 2007), tiger frog virus (Guo et al., 2011) and BK virus (Eash et al., 2004).

A previous study demonstrated high levels of expression of nectin-4 in sheep epithelial tissues taken from the mouth, upper respiratory tract and stomach (Birch et al., 2013). The high levels of expression of nectin-4 in epithelial tissues are supportive of a role for this protein in PPRV epithelial pathogenesis. Disease in sheep and goats is often associated with serious pathology in the intestine (Couacy-Hymann et al., 2007; Hammouchi et al., 2012); however, the level of nectin-4 in these tissues is relatively low (Birch et al., 2013). Moreover, although high levels of nectin-4 expression were detected in the stomach, pathology in this organ is rare (Couacy-Hymann et al., 2007; Hammouchi et al., 2012; Birch et al., 2013). Importantly, PPRV can cause abortion in pregnant sheep and goats (Borel et al., 2005; Abubakar et al., 2008). Interestingly, our data found that EECs can also express a nectin-4 receptor, and the levels of goat nectin-4 correlated well with PPRV infection *in vitro*. However, examination of cell cultures *in vitro* may not accurately reflect the situation *in vivo*, and therefore further research is needed in this area. In addition, a recent study reported that MeV can upregulate nectin-4 expression in human and murine brain endothelial cells (Abdullah et al., 2013). Our findings revealed that the level of nectin-4 was significantly increased following PPRV infection in EECs but not in FFCs, indicating that PPRV entry and infection plays an important role in regulating the expression of nectin-4 in host epithelial cells. However, PPRV infection did not induce the expression of nectin-4 in FFCs. These data indicated that PPRV may not use nectin-4 as a receptor in FFCs and probably enters cells via a different route. Previous studies have shown that some viruses can actually utilize one or more endocytic pathways to enter host cells since the regulation of endocytosis is pleiotropic and cell type dependent (Cossart and Helenius, 2014). Our results also demonstrated that PPRV enters FFCs through a dynamin-dependent but not a clathrin- or caveolae-mediated endocytosis pathway, indicating that the requirements for PPRV endocytosis were cell type specific. Overall, we showed that not all inhibitors tested could completely block PPRV entry and replication, suggesting that PPRV may use more than one pathway for entry into different cells, i.e., cell fusion and the endocytic pathway.

For a virus to activate an efficient infection, it must first gain access to a cellular receptor, which may be pH-dependent and -independent (Nicola et al., 2003; Zhu et al., 2012; Huang et al., 2015). The internalization of paramyxovirus was reported to require an acidic pH, and an endosomal low pH triggered a series of intracellular molecular events (San Roman et al., 1999; Sanchez-Felipe et al., 2014). In this study, exposure to an acidic pH appeared to be a requirement for successful entry and infection of EECs by PPRV. Our results showed that PPRV entry into EECs was inhibited by an elevated pH in the intracellular acidic compartments. This was in contrast to FFCs, in which PPRV entry was achieved primarily at neutral pH, potentially resulting in differences in subsequent signal pathways.

Dynamin promotes membrane fission to generate endocytic vesicles during several endocytic pathways (Zhu et al., 2012). However, the role of dynamin in the internalization process of PPRV is poorly understood. In this study, our results indicated that dynamin plays an essential role in the caveolae-mediated endocytosis of PPRV into EECs. Although dynamin activity was thought to be specific to caveolae- and clathrin-mediated endocytosis pathways (Damke et al., 1994), our data indicated the essential role of dynamin in the internalization of PPRV into FFCs and this effect was independent of clathrin and caveolin. This finding also confirmed the essential role of dynamin in membrane fusion (Damke et al., 1994; Low and Lowe, 2010) and that dynamin is necessary for the internalization of numerous viruses (Perry and Wobus, 2010; Guo et al., 2011; Rahn et al., 2011). In addition, membrane cholesterol is a component of the lipid raft and plays important roles in the caveolin-mediated endocytic pathway (Riezman et al., 1997; Mercer and Helenius, 2009). The reorganization of the membrane lipid raft significantly inhibited PPRV internalization and multiplication. Therefore, the depletion of cholesterol by treatment with MβCD likely rearranged the phosphoinositide in the cell membrane, thereby blocking the binding domain for internalization.

An increasing number of viruses have been found to utilize the macropinocytosis pathway to enter permissive cells (Coyne et al., 2007; Mercer and Helenius, 2008; Kalin et al., 2010; de Vries et al., 2011; Delpeut et al., 2017). In the present study, when the macropinocytosis pathway was blocked with EIPA, the efficiency of PPRV infection did not decrease. This may indicate that macropinocytosis offers a non-infective pathway of entry for PPRV into host cells. In addition, a number of recent studies demonstrated that viruses may also take advantage of the PI3K-AKT pathway when entering cells via endocytic pathways (Mercer and Helenius, 2008; Sanchez et al., 2012). Our present data showed that blocking PI3K activity not only inhibited PPRV initial entry, but also arrested viral replication. These results suggested that PI3K may regulate the caveolin-mediated endocytic pathway by triggering complex signaling pathways. Wortmannin has also been reported to inhibit members of the polo-like kinase family (Liu et al., 2005) and to be a highly selective inhibitor of PI3K-related kinases, such as mTOR, ATR and the catalytic subunit of DNA-dependent protein kinase (Brunn et al., 1996; Wymann et al., 1996; Walker et al., 2000). Our data further imply that there is a close correlation between PPRV and various kinases, although the precise mechanism remains to be elucidated. Moreover, recent studies indicated that PPRV exploits the cellular autophagy machinery for replication (Richetta et al., 2013; Zhang et al., 2013). The inhibition of PI3K activity may have inhibited early autophagy, thereby reducing virus entry and replication.

## Conclusion

The present study provides a systematic model with which to identify the entry mechanism of PPRV into host cells. Our data revealed that PPRV enters EECs in a cholesterol-, pH- and dynamin-dependent manner via the caveolae-mediated endocytosis pathway, accompanied by PI3K regulation. Demonstrating the viral and cellular components involved in PPRV entry into host cells, combined with the underlying mechanisms that govern this process, should provide insight into new antiviral therapeutic strategies. This work might contribute to the development of novel methods to control and prevent the spread of PPRV and other paramyxoviruses.

## Author Contributions

BY carried out the experiments, collected data, and wrote this manuscript. XQ checked and revised the manuscript. HG, PJ, SC, ZC, and TW participated in some of the experiments. JW and QX conceived the study and participated in its design and coordination. All authors discussed the results, commented on the manuscript, and approved the final version.

## Conflict of Interest Statement

The authors declare that the research was conducted in the absence of any commercial or financial relationships that could be construed as a potential conflict of interest.
